# Posterolateral Corner Reconstruction Alone Using a Fibular-Based Technique in a Patient with Persistent Unstable Revision Total Knee Arthroplasty

**DOI:** 10.1155/2015/262187

**Published:** 2015-12-31

**Authors:** Joseph T. Cline, Eduard Alentorn-Geli, J. H. James Choi, Joseph J. Stuart, Terry Kruger, Claude T. Moorman III

**Affiliations:** ^1^University of North Carolina School of Medicine, 321 S. Columbia Street, Chapel Hill, NC 27516, USA; ^2^Duke Sports Sciences Institute, Department of Orthopaedic Surgery, Duke University, 3475 Erwin Road, Durham, NC 27705, USA

## Abstract

Posterolateral rotatory instability is a relatively uncommon cause of unstable total knee arthroplasty (TKA). In most cases, surgical treatment requires revision TKA into a more constrained design or thicker polyethylene liner. We present a case of a patient with unstable TKA who remained unstable after increasing thickness of the polyethylene liner and undergoing more constrained TKA. After several revision surgeries, the patient was still unstable. Posterolateral corner reconstruction with a fibular-based technique using a tibialis anterior allograft was performed. At 1-year follow-up, the patient was stable and asymptomatic and with excellent function. A soft-tissue procedure only (fibular-based posterolateral corner reconstruction) can be effective at restoring posterolateral rotatory stability in a patient with persistent instability after revision TKA.

## 1. Introduction

As per Centers for Disease Control statistics, there were 719,000 knee arthroplasties performed in the United States in 2010 [1]. The success of total knee arthroplasty (TKA) can be measured by the rate at which this surgical procedure requires reoperation. The revision rate in posterior cruciate ligament-retaining TKA over 27 years has been reported to be 0.4% per year [2]. Revision secondary to ligamentous instability accounts for roughly 5% of TKA [2]. Posterolateral corner (PLC) instability is a rare problem after TKA but can lead to significant morbidity and implant failure. There is little reported data on the management of this condition and none of the current studies address the efficacy of soft-tissue reconstruction alone in the treatment of these patients who have failed revision to a more constrained design. In one study of 44 patients receiving a condylar constrained knee design due to ligamentous laxity or severe varus/valgus deformity, 7% of the primary arthroplasty and 13% of the revision arthroplasty patients were considered to have poor results by the Tegner Lysholm Knee Scoring Scale [3]. Comparing patients undergoing revisions with these condylar constrained designs due to varus/valgus instability with patients receiving posterior stabilized revisions without instability, studies have found similar results in terms of long term satisfaction and survivorship of the implant [4]. However, none of these studies address treatment for patients with suboptimal outcomes beyond further revision.

The purpose of this case report was to describe a case of persistent posterolateral instability after several revision TKA surgeries, including revision to a condylar constrained design, satisfactorily corrected with soft-tissue reconstruction alone (fibular-based PLC reconstruction using a tibialis tendon allograft) without further prosthesis revision.

## 2. Case Presentation

A 59-year-old female with left TKA was referred to our clinic complaining of left knee pain and instability. She had an extensive orthopedic history as a result of significant bilateral tricompartmental arthritis unresponsive to conservative management, including a reported history of 10 surgeries on her right knee related to TKA. She had undergone a left TKA 6 years earlier using a PCL-sparing prosthesis (Zimmer Natural-Knee gender specific high flexion knee system, Zimmer Inc., Warsaw, IN, USA). Following this procedure, she had recurrent sharp lateral joint pain during activity, requiring arthroscopic lysis of adhesions after 1 year. At that time, she did not experience any issues with instability. Twenty months postoperatively, she was noted to have 120 degrees of flexion with good varus-valgus stability but continued to have pain. She underwent revision TKA 22 months after the index procedure and intraoperatively was found to have lateral impingement due to scar tissue along with loose tibial and patellar components. At this setting, she underwent debridement of scar tissue with synovectomy and revision of femoral and tibial components. This revision lasted 34 months, before atraumatic dislocation of the polyethylene insert after standing from a seated position prompted another revision. In this procedure, she was revised to a condylar femoral constrained prosthesis, the NexGen Legacy Constrained Condylar Knee (LCCK), Zimmer (Zimmer Inc., Warsaw, IN, USA). This prosthesis is designed to limit varus/valgus movement to 1.25 degrees and internal/external rotation to 2 degrees. After 10 months with this condylar constrained prosthesis, she again experienced dislocation of the polyethylene insert. At that time, she underwent another revision replacing her 19 mm polyethylene insert with a 22 mm insert due to lateral collateral ligament (LCL) laxity that was documented intraoperatively. This thicker liner was observed intraoperatively to stabilize the joint without altering the knee's mechanics and range of motion. Despite this change, she experienced another dislocation of her polyethylene liner only 5 months after her revision. She underwent her fourth revision, and after the knee could not be reduced with a trial of a 25 mm liner, a 22 mm liner was again used. Following this revision, she continued to experience instability with minimal activity and was referred to our clinic for evaluation for PLC instability and possible ligament reconstruction.

**Figure 1:**
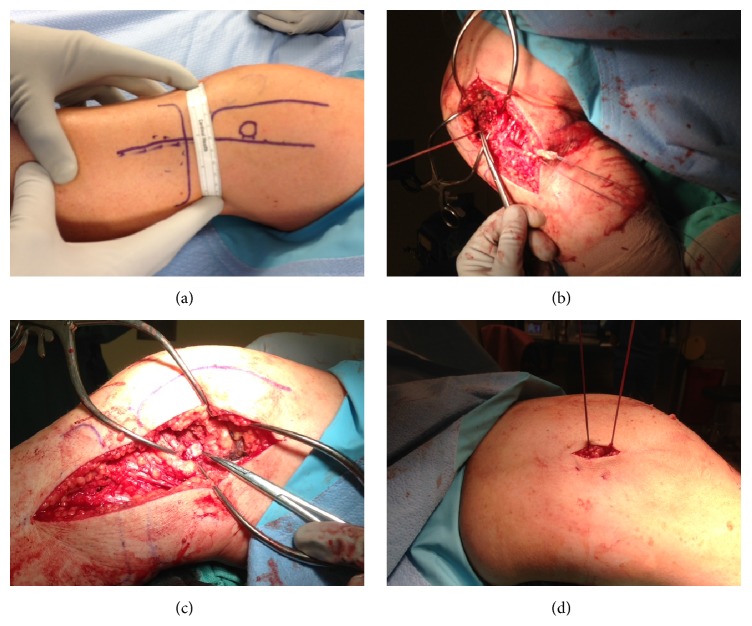
Intraoperative pictures demonstrating the surgical technique. (a) Minimum 7 cm distance between the anterior skin incision (from previous surgical procedures) and the new incision for the posterolateral corner injury. (b) The tibialis anterior allograft has been passed through the proximal fibula, and a Beath pin has been placed in the desired location for the femoral attachment (pointed by the Metzenbaum scissors). (c) Final appearance of the allograft fixed with the screw in the lateral aspect of the distal femur. (d) Medial view of the knee demonstrating the passage of the 2 Beath pins through the femoral socket to create two independent tunnels. The pins are used to pass a suture in each one to create the additional aperture fixation in the medial side.

**Figure 2:**
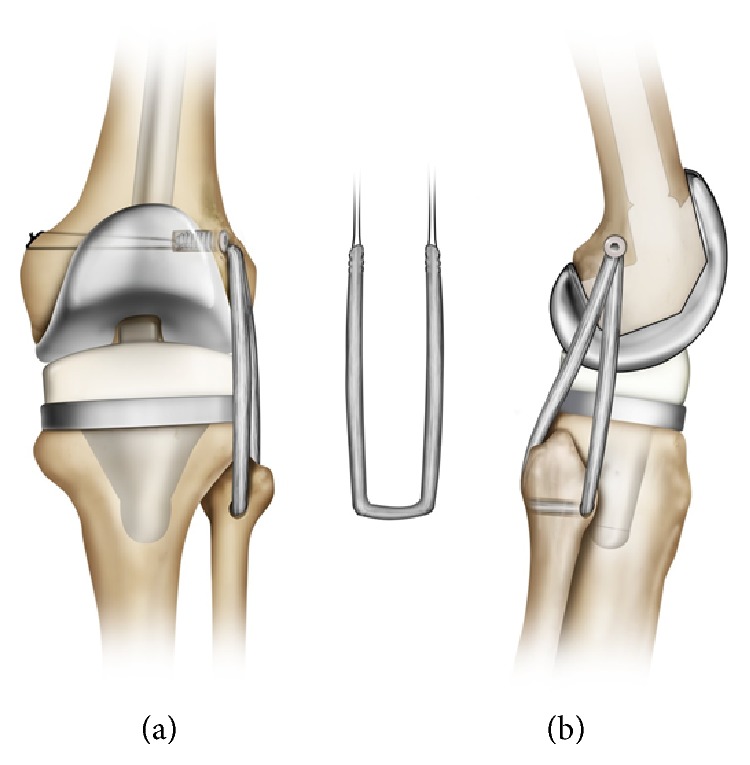
Representation of the fibular-based posterolateral corner reconstruction with tibialis anterior allograft and its relationship to the condylar femoral constrained prosthesis.

On exam, she was noted to have significant LCL laxity, especially in flexion, and increased posterolateral rotational instability (positive dial test), consistent with PLC instability. The patient was scheduled for PLC reconstruction to restore stability to her knee. This reconstruction was accomplished by a modified version of the fibular-based technique described by Larsen et al. [5] (Figures 1 and 2). A tibialis anterior allograft was used instead of the described semitendinosus graft, and no figure eight loop was incorporated into the reconstruction. The allograft was passed through a single 7 mm fibular tunnel created in a slightly oblique anterolateral to posteromedial direction (Figures 1 and 2). Then, a single tunnel of 9 mm diameter and 30 mm depth was created at the femoral site and both ends of the graft were attached at the same femoral site with a Milagro Advance (Depuy Synthes Mytek, Raynham, MA, USA) interference screw (Figures 1 and 2). Two separate Beath pins were drilled lateral to medial to the femoral socket to create two independent tunnels. Then, the sutures placed at each end of the graft were passed through these lateral-to-medial tunnels to create aperture fixation in the medial side (Figures 1 and 2). The graft was tensioned into place with the knee in 30 degrees of flexion, slight valgus stress, and internal rotation of the foot. Intraoperatively, there was no observable varus or posterolateral instability following placement of the graft.

At 1-year follow-up, the patient had no pain or subjective instability. She had pain in the anterior thigh and limited knee flexion for several months, which significantly improved with deep tissue massage into the distal quadriceps area. The range of motion was 5° loss of extension to 120° of flexion. The varus stress test revealed no lateral opening and the dial test was negative. The patient had a score of 54.8 for the Physical Component Summary and 59.8 for the Mental Component Summary of the SF-12. The patient had a Knee Score of 83 and a Function Score of 100 in the Knee Society Score. On a 0-to-10 satisfaction scale where 10 is the maximal satisfaction, the patient rated her surgical treatment as 10.

## 3. Discussion

PLC instability presents a rare but potentially significant problem for patients following TKA. The methods of addressing instability can be as conservative as using a brace or involve surgical options such as revision to a thicker liner, a more constrained prosthesis, a hinged prosthesis, or, in the most extreme cases, an arthrodesis [6]. Previous studies have shown that patients with ligamentous laxity receiving revision with a constrained design have similar long term functional outcomes and prosthesis survivorship to patients without ligamentous instability receiving posterior stabilized implants [4, 7]. The constrained prosthesis used in this case was one designed to limit varus/valgus and rotational motion but was insufficient on its own at eliminating the instability the patient experienced. As was presented in this case, soft-tissue reconstruction of the PLC with a modified fibular-based technique alone presents a viable option for patients who have instability that persists after revision TKA to a thicker polyethylene liner and condylar constrained design prosthesis.

While the number of studies covering approaches to lateral ligamentous instability following TKA is limited, there have been several methods described in the literature. Unnanuntana et. al. published one such report on managing lateral TKA laxity with LCL reconstruction [6]. They describe a patient who had failed a constrained design prosthesis due to LCL deficiency and subsequently underwent a revision TKA with concurrent LCL reconstruction. Ultimately, this trial failed before its success could be determined due to infection, and a rotating-hinge knee prosthesis had to be used [6]. Ohnsorge et al. reported on LCL reconstruction with simultaneous revision of TKA to a condylar constrained design with a good outcome [8]. Additionally, there has been PLC reconstruction after TKA described by Flierl et al. following a similar modified Larsen technique to the one described in this case [9]. Along with the reconstruction, the original polyethylene insert was replaced with a deep dish, larger polyethylene insert to provide more stability to the knee [9]. A noticeable commonality among all the existing cases that have been reported is the use of some revision of the TKA to a more constrained design to support the ligamentous reconstruction. It may be difficult to determine whether patients saw more benefit from the reconstruction or the use of a new constrained prosthesis.

Medial instability after TKA has also been documented. Pritsch et al. reported a series of seven patients who all had failures of medial collateral ligament- (MCL-) stabilizing procedures performed following medial instability in the setting of recent TKA [10]. These patients were followed up for 10 years, with failure of the reconstruction occurring as late as 1 year after surgery. Two patients experienced reconstruction failure immediately after removing immobilization, with the average time to failure being approximately 13 weeks. Of note, only one patient's MCL reconstruction lasted longer than 3 months. Following these results, they concluded that ligamentous reconstruction alone was insufficient to support an unstable TKA and recommended that any instability be addressed with soft-tissue reconstruction and revision either to a larger insert component or to constrained design prosthesis [10]. In our case, the patient had already failed a condylar constrained design twice with atraumatic dislocation of the polyethylene liner with activities of daily living and was unable to tolerate a larger insert (she already had a 22 mm liner) to remove laxity. Since the PLC reconstruction, she has had no further dislocations of her prosthesis and has been very pleased with the outcome. While medial instability may not be correctable with ligamentous reconstruction alone, our results indicate that posterolateral corner instability following TKA can be addressed without the need for revision surgery to more restricted knee designs.

In conclusion, this case report has shown that a case of persistent PLC instability after TKA treated with revision to a thicker polyethylene liner and a more constrained design can be solved by soft-tissue reconstruction alone by means of allograft PLC reconstruction using a fibular-based technique.
